# OMEinfo: global geographic metadata for -omics experiments

**DOI:** 10.1093/bioadv/vbae025

**Published:** 2024-02-21

**Authors:** Matthew Crown, Matthew Bashton

**Affiliations:** Hub for Biotechnology in the Built Environment, Department of Applied Sciences, Faculty of Health and Life Sciences, Northumbria University, Newcastle upon Tyne, NE1 8ST, United Kingdom; Hub for Biotechnology in the Built Environment, Department of Applied Sciences, Faculty of Health and Life Sciences, Northumbria University, Newcastle upon Tyne, NE1 8ST, United Kingdom

## Abstract

**Summary:**

Microbiome studies increasingly associate geographical features like rurality and climate with microbiomes. It is essential to correctly integrate rich geographical metadata; and inconsistent definitions of rurality, can hinder cross-study comparisons. We address this with OMEinfo, a tool for automated retrieval of consistent geographical metadata from user-provided location data. OMEinfo leverages open data sources such as the Global Human Settlement Layer, and Open-Data Inventory for Anthropogenic Carbon dioxide. OMEinfo's web-app enables users to visualize and investigate the spatial distribution of metadata features. OMEinfo promotes reproducibility and consistency in microbiome metadata through a standardized metadata retrieval approach. To demonstrate utility, OMEinfo is used to replicate the results of a previous study linking population density to bacterial diversity. As the field explores relationships between microbiomes and geographical features, tools like OMEinfo will prove vital in developing a robust, accurate, and interconnected understanding of these interactions, whilst having applicability beyond this field to any studies utilizing location-based metadata. Finally, we release the OMEinfo annotation dataset of 5.3 million OMEinfo annotated samples from the ENA, for use in retrospective analyses of sequencing samples, and suggest several ways researchers and sequencing read repositories can improve the quality of underlying metadata submitted to these public stores.

**Availability and implementation:**

OMEinfo is freely available and released under an MIT licence. OMEinfo source code is available at https://github.com/m-crown/OMEinfo/ and https://doi.org/10.5281/zenodo.10518763

## 1 Introduction

The microbial ecology of the built environment plays a role in shaping our human health (Kirjavainen *et al.* 2019), and the environment around us, e.g. through microbially induced corrosion (Salgar-Chaparro Silvia *et al.* 2020). Recently, much focus has been placed on associating geographical features such as rurality (Parajuli *et al.* 2018), climate type, and even specific cities (Danko *et al.* 2021) with microbiomes. For example, Wang *et al.* (2018) demonstrate a positive correlation between population and soil bacterial diversity. As the evidence for such associations has grown, so has the size of studies being performed to examine these effects (Danko *et al.* 2021). With global-scale studies, comes a need for globally consistent metadata annotation, for both consistency within a study, as well as for meta-analysis of global studies.

These projects require the determination of geographical metadata features, typically through online data sources such as Google Maps for latitude, longitude, and elevation or GIS services such as ArcGIS Online. Microbiologists/bioinformaticians are typically not familiar with the plethora of geographical data available publicly through e.g. satellite-derived GeoTIFFs, and even so, may not be aware of the tools and methods required to integrate these into their analysis. This has led to many studies in the field employing an ambiguous, approximate or sub-optimal definition of many environmental characteristics, including rurality (Kirjavainen *et al.* 2019) and climate classification (Chase *et al.* 2016), limiting the ability to compare between studies and re-analyse data in a global context (see Supplementary Table S1 for a more comprehensive list of differences between study definitions of such metadata features and their issues).

In addition, some measurements, such as rurality, are often determined by an assumed value for a location or through a determination made by local authorities or governments. This can lead to issues when comparing across studies, where definitions may vary significantly—i.e. what is considered rural in a densely populated country may be considered urban in another country see Supplementary Table S2 for examples of these.

To be consistent in the use of geographical metadata, it is therefore crucial that the source of metadata features is communicated, something which is often overlooked, and where possible, globally consistent definitions for these features are used. To date, no tool exists to provide such geographical/cultural/socioeconomic metadata feature annotation in a globally consistent manner. To this end, we present OMEinfo, a tool for the automated retrieval of consistent geographical metadata including Köppen-Geiger climate classification, degree of rurality, population density, CO_2_ and NO_2_ emissions and relative deprivation from user-provided location data, together with a versioned data source which allows for metadata provenance.

## 2 Implementation

**Figure 1.**
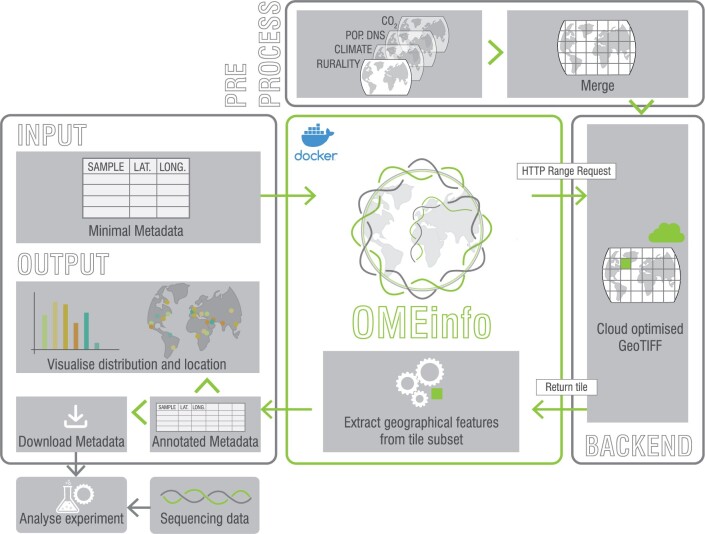
The OMEinfo workflow. Users upload a metadata file with sample, latitude, and longitude to the Dockerised web app (or CLI). OMEinfo processes locations and retrieves relevant sub-tiles from the Cloud Optimized GeoTIFF necessary for annotating the location. Features are annotated for each location and upon completion, interactive maps and plots are displayed to the user. Metadata and citations can then be downloaded and integrated into downstream analysis. This workflow is further described in Sections 2.1 and 2.2. A walkthrough of using the Dash app is provided in Supplementary Information Section 2.

OMEinfo is released as a Dash based web app, designed to run locally in the browser. An overview of the OMEinfo workflow can be seen in Fig. 1. More information about data sources and implementation are given below (Sections 2.1 and 2.2) and in Supplementary Information Section 1.

### 2.1 Data sources

OMEinfo utilizes cloud-optimized GeoTIFFs (COGs) for back-end data storage, available online in FigShare. Various data sources are integrated into the OMEinfo v2 dataset covering global geographical, environmental, and socioeconomic characteristics (see Supplementary Table S3 for details and the approximate update schedule of these underlying data sources). Once released, each data packet will remain static for backwards compatibility, with new underlying data updates integrated into separate new COG format data packets. See supplementary information Section 1 for a description of the data aggregation and preprocessing procedure.

### 2.2 OMEinfo tool implementation

OMEinfo is available as both a Dockerised web-app (OMEinfo app) and a command-line tool (OMEinfo CLI). OMEinfo app is built with Dash v2.9.3 (dash: Data Apps & Dashboards for Python. No JavaScript Required n.d.). OMEinfo CLI provides a convenient method for power users to process data directly in the command-line, allowing for batch processing and integration into pipelines, and additionally makes use of Rich v13.6.0(rich: Rich is a Python library for rich text and beautiful formatting in the terminal, n.d.) for rich text output in the terminal. Both the CLI and app require users to upload a metadata sheet including sample name, latitude, and longitude in CSV/TSV format. Latitude and longitude should be in the common EPSG : 4326 coordinate system, which is a format used by all common web-based mapping tools, e.g. Google Maps and OpenStreetMap.

OMEinfo utilizes rio-cogeo (rio-cogeo: Cloud Optimized GeoTIFF creation and validation plugin for rasterio, n.d.) to query the COG files. Queries take the form of HTTPS range requests, which return small, but not uniquely identifiable, subsets of the OMEinfo COG locally, where they are queried for specific point data. Rio-cogeo supports query caching, meaning that if locations are geographically similar, locally cached data storage can significantly speed up annotation. Additionally, by submitting HTTPS range requests rather than transmitting the locations themselves, OMEinfo keeps sensitive location metadata private, helping to keep personally identifiable information secure, and allowing for metadata to be distributed with globally consistent metadata features, whilst maintaining anonymization of data. This feature benefits studies in which ethical approvals prevent the sharing of location data, for example, sampling in an individual’s home. A full list of software utilized in the development of OMEinfo, its version, and function is available in Supplementary Table S4.

Following analysis completion, OMEinfo app presents users with a summary of annotated metadata fields, in the form of interactive histograms, maps and tables, which allows users to see the OMEinfo metadata in context.

OMEinfo is committed to the principles of FAIR data sharing. Code and data sources essential for constructing OMEinfo data packets are openly accessible (see Data availability section). All code is released under the permissive MIT licence, and the GeoTIFF data utilized within OMEinfo adheres to permissive data usage licences, as specified in Supplementary Table S2. We prioritize interoperability by citing all data sources employed within the application in a downloadable BibTeX format, both within the web application and the repository. By versioning the formatted data packets upon release and documenting their contents in a changelog within the repository, we ensure that previous data versions remain accessible within FigShare (Data availability section) for continued use, encouraging data reusability and promoting sustainability in research efforts.

## 3 Applications and examples

### 3.1 Potential use cases

OMEinfo is usable in any study employing sampling which can be associated with a specific geographic location. It allows for automated and consistent metadata annotation based on these locations. The tool was originally developed for use with the built environment metagenomics analysis (or any -omics data, hence OME) with potential use cases including:

- Determining associations between microbiome diversity and geographical metadata features, e.g. population density (Section 3.2).
- As a ground truth dataset for classification algorithms utilising microbial taxa abundances for location classification.
- To identify locations with specific environmental conditions suitable for microbial biotechnological applications (e.g. bioprospecting for extremophiles in areas with high NO_2_ concentrations).

### 3.2 Correlation between OMEinfo-derived and census-derived metadata

Wang *et al.* (2018) utilize population density estimates derived from US census data and census tract blocks to determine a population density/km^2^ and show a positive relationship between population density and bacterial diversity. To verify the accuracy of population density annotation from OMEinfo (in v2 data packet at 1 km^2^ resolution), Spearman's rank correlation analysis was used to assess the relationship between OMEinfo population densities and the original study’s census-tract defined population densities. The results revealed a strong positive and statistically significant correlation (Spearman's ρ = 0.78, *P* < .01, Supplementary Fig. S1).

Some variability between census-tract and OMEinfo-derived values is observed at higher population densities (Supplementary Fig. S1). This is likely due to the difference in method for estimating population density: in the Global Human Settlement Layer Population GeoTIFF, a population disaggregation model is used to distribute the underlying population to built-up areas, whereas a census can precisely determine the population living in a given area. However, the benefit of the OMEinfo approach to assigning population density remains, in that from a single web app/CLI tool, population density, together with a host of additional metadata features, can be determined globally and consistently without needing to obtain individual census data, making it well suited to providing consistent metadata for complex global studies.

To verify the applicability of OMEinfo in a biological context, Spearman’s rank correlation was utilized to re-evaluate the finding previously reported in the same study, in which population density showed a positive association with Shannon diversity and phylogenetic diversity. A positive relationship was observed between Shannon diversity and population density, using OMEinfo-derived population density and Census Tract population density, as used in the original study (Wang *et al.* 2018) (Original Study Data: Spearman Correlation Coefficient 0.225, *P* < .01, OMEinfo: Spearman Correlation Coefficient 0.214, *P* < .01, see Supplementary Fig. S2) Similarly, Phylogenetic Diversity exhibited a correlation of 0.259 with Census Tract Population Density and 0.244 with OMEinfo Population Density, both statistically significant (*P* < .01).

### 3.3 OMEinfo annotation dataset

The European Nucleotide Archive (ENA) (Burgin *et al.* 2023) sequence read archive serves as a central repository of read data. As part of submission to ENA, users may choose to distribute location data for associated biosamples. OMEinfo was utilized to produce two datasets annotated with globally consistent geographical metadata: OMEinfo ENA v1 (5.3million samples) and OMEinfo ENA Env v1 (135 517 samples marked environmental) taking less than 30 and 1 min, respectively. See Supplementary Information Section 2 for details on the performance of OMEinfo on large datasets.

**Figure 2.**
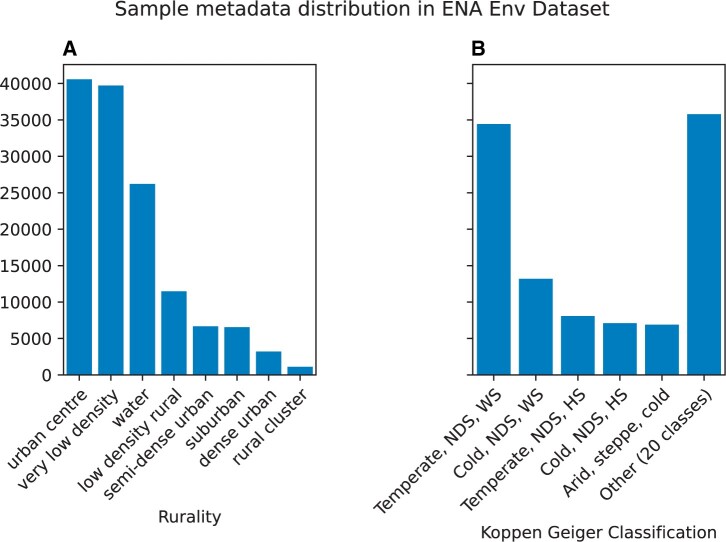
Sample metadata distribution in the ENA Env. dataset. (A) The breakdown of samples according to the rurality index shows most samples are from urban centres, very low-density locations or water. (B) Breakdown of samples according to Köppen-Geiger Climate classification. Most samples are taken from ‘Temperate, no dry season, warm summer’ climates. NDS, no dry season; WS, warm summer; HS, hot summer.

Using these datasets, we find that environmental samples in the ENA show a skew towards the extremes of the urban-rural scale (Fig. 2A), with 78.59% of all environmental samples annotated as urban centres (40 565 samples), very low density (39 716 samples), or water (26 216 samples). Future global studies of rurality/urbanization should ensure that focus is given to all levels of the urban-rural continuum, as it is possible that additional insights could be gained by sampling from understudied areas of such a continuum.

This pattern is even more pronounced for Köppen-Geiger climate classifications, where 25.41% of all environmental samples are from locations with a ‘*Temperate, no dry season, warm summer*’ climate type (Fig. 2B). This is especially relevant as other studies have shown that climate type is a driver of microbiome diversity in the built environment (Danko *et al.* 2021).

Within the full ENA dataset, more than 3700 samples report a location of 0.00N 0.00E for their sample, a location in the South Atlantic Ocean. Samples with this location also have ‘Environment Biome’ metadata values including the following:

- Wetland
- Human-gut
- International Space Station—Russian Service Module
- Anthropogenic forest
- Large river biome

From this, it is clear that certain locations, including 0.00N 0.00E, are used as placeholders to satisfy metadata requirements. Within the environmental database, we can see several frequent sample locations occurring. Whilst some of these represent genuine sample locations, e.g. a site in De Mossel (NL), where long-term experiments have been occurring, many others misrepresent the location reported in studies themselves, for example, 1341 samples from SARS-CoV-2 wastewater sequencing in Liverpool (UK) have a reported location in the North Sea.

To this end, we provide a set of recommendations for submitters to ENA of additional best practices, on top of following existing metadata standards, for submitting data to public read repositories in order to provide the greatest value to downstream users of this data:

- Avoid use of pseudo latitude and longitudes for samples where this data is not available.
- Submit locations at the maximum possible resolution allowable by ethical approvals. In most cases, data can be collected at a high resolution using a modern smartphone, for example with Google Maps or What3Words, and easily converted to latitude and longitude for upload.
- Where sample locations are not able to be shared, use International Nucleotide Sequence Database Collaboration (INSDC)-recommended controlled vocabulary to report this, e.g. ‘not applicable’, ‘not collected’, ‘not provided’, or ‘restricted access’.

Further, whilst public sequence read repositories such as the ENA have made encouraging updates to minimum metadata standards, including requiring the country of collection for all new samples, implementing additional checks for consistency between metadata fields upon submission could ensure the quality of data stored is not avoidably diminished, particularly improving the geographical remediation of metadata records. For example, with these new collection country metadata requirements, it is possible to determine whether coordinates correlate with the reported location and flag this to the user. Additionally, it is possible with OMEinfo to determine if sample coordinates are on a body of water, which can then be sanity-checked against reported ‘Environment Biome’ metadata fields.

Finally, we observed that 22 626 samples had invalid latitude and longitude metadata fields within the full ENA dataset. Except for checklists ERC000028 and ERC000011 (the ENA default sample checklist), where lat_lon is an optional free text field, all location fields are in the WGS84 decimal degrees format, with submission controlled by the following regular expression: (^[+−]?[0–9]+.?[0–9]{0,8}$). In WGS84 decimal degree format, latitudes should be between ±180° and longitudes between ±90°. However, valid strings for this regular expression include 12 345 and 1234567890a12345678, and we observe latitude and longitudes orders of magnitude greater than possible in WGS84 coordinate system, e.g. 645209.099648 and 11500.859. To check for valid latitudes and longitudes, we suggest the ENA move to two separate regular expressions, one for latitude and one for longitude:

Latitude: (^[-+]?([1-8]?\d(\.\d+)?90(\0.0+)?)$)

Longitude: (^[-+]?((1[0-7]\d(\.\d+)?)|([1-9]?\d(\.\d+)?)|180(\0.0+)?)$)

These regular expressions limit latitude to between ±90.0 and longitude to between ±180.0, whilst allowing any level of granularity in the decimal fraction.

We recommend consolidation of all location-based checklist fields to the WGS84 decimal degree format, and enhanced validation of submitted metadata to ensure that latitudes and longitudes fall within the expected bounds of the coordinate system. Such remediation will help to improve the quality of submitted data and ensure communication of geolocation is clear and consistent for samples in the ENA regardless of their chosen checklist.

## 4 Conclusion

We present a powerful, innovative tool for annotating metadata with essential geographical features using FAIR data. This solution not only enhances the granularity of microbial sequencing metadata but also opens up new possibilities for understanding the interplay between microorganisms and their environments. OMEinfo has broad applicability beyond microbial ecology to any dataset with location-based features. To enable researchers to begin using such data, we present ‘OMEinfo ENA’ and ‘OMEinfo ENA Env.’ datasets; metadata annotations of all samples in the ENA with reported locations, as well as the OMEinfo tool in a pre-built Docker container and a simple CLI tool.

## Supplementary Material

vbae025_Supplementary_Data

## Data Availability

OMEinfo is freely available and released under an MIT licence. OMEinfo source code is available at https://github.com/m-crown/OMEinfo/ and is archived in Zenodo (DOI: https://doi.org/10.5281/zenodo.10518763). The OMEinfo v1 data packet is available at https://doi.org/10.6084/m9.figshare.25000025.v1 and the OMEinfo v2 data packet at https://doi.org/10.6084/m9.figshare.25000343.v1. OMEinfo ENA and OMEinfo ENA Env Data analyses are available on FigShare (DOI: https://doi.org/10.6084/m9.figshare.25003217.v1).
