# Supplementary material for: OMEinfo: global geographic metadata for -omics experiments
Source: Bioinform Adv. 2024 Feb 21;4(1):vbae025. doi: 10.1093/bioadv/vbae025 (PMC10918632; doi:10.1093/bioadv/vbae025)
Supplement: vbae025_Supplementary_Data [file vbae025_supplementary_data.docx]

# **Supplementary Data**

### **Supplementary Information Section 1: Expanded Implementation**

### **Data Aggregation and Preprocessing**

Fossil fuel CO2 emission data from ODIAC is averaged using the average_geotif.py script in OMEinfo repository (see <https://doi.org/10.5281/zenodo.10518764>). Tropospheric NO2 data is averaged using Google Earth Engine (see data_processing.md in the OMEinfo repository for code)

All data sources are reprojected into World Geodetic System 1984 (WGS84/ESRI:4326) format and merged into a single multi-band file using Geospatial Data Abstraction Library (GDAL) v3.7.0[(GDAL/OGR contributors, 2023)](https://paperpile.com/c/zaN4J5/UnZlj). The multi-band WGS84 projection file is then converted to COG format using Rasterio v1.3.7[(Gillies and Others, 2013-)](https://paperpile.com/c/zaN4J5/MYPRY) and the rio-cogeo v5.0 plugin[(*rio-cogeo: v5.0.0*, no date)](https://paperpile.com/c/zaN4J5/cCsoA).

This COG file is then uploaded to FigShare and given a citable version specific DOI (v1 DOI: <https://doi.org/10.6084/m9.figshare.25000025.v1>, v2 DOI: <https://doi.org/10.6084/m9.figshare.25000343.v1>), and is queried by the OMEinfo web-app, but can also be downloaded by end-users should they wish to interact directly with the data sources utilised in OMEinfo.

**Location Query Implementation in OMEinfo Tool (App + CLI)**

Under the hood, OMEinfo utilises rio-cogeo, rasterio and GDAL to annotate uploaded locations with globally consistent geographic metadata.

1. A Reader is created pointing to the URL of the OMEinfo data packet.
2. OMEinfo then iterates over each uploaded location and queries the location using the rio-cogeo “point” function.
   In the underlying OMEinfo data packet, each metadata field e.g. Rurality, Population Density, Climate Type, is represented by a separate layer of the geoTIFF.
   1. The “point” function converts the latitude/longitude to a pixel value in the image.
   2. The relevant tile from the geoTIFF is then requested using HTTPS range requests by rio-cogeo.
   3. From this tile, the pixel values representing the location are accessed and the individual metadata values are stored in variables.
3. Upon completion of iteration through locations, the DataFrames are then visualised, either through maps and histograms/bar charts within the app or through a table in the command line tool. See supplementary section 2 for more information on these visualisations.

### **Supplementary Information Section 2A:** a walkthrough of the OMEinfo workflow using the Dash-app.

1. Upon launching the tool, users are greeted with a splash page detailing some of the data and features of OMEinfo. To begin analysis, click the analyse button, or navigate to the analyse page.


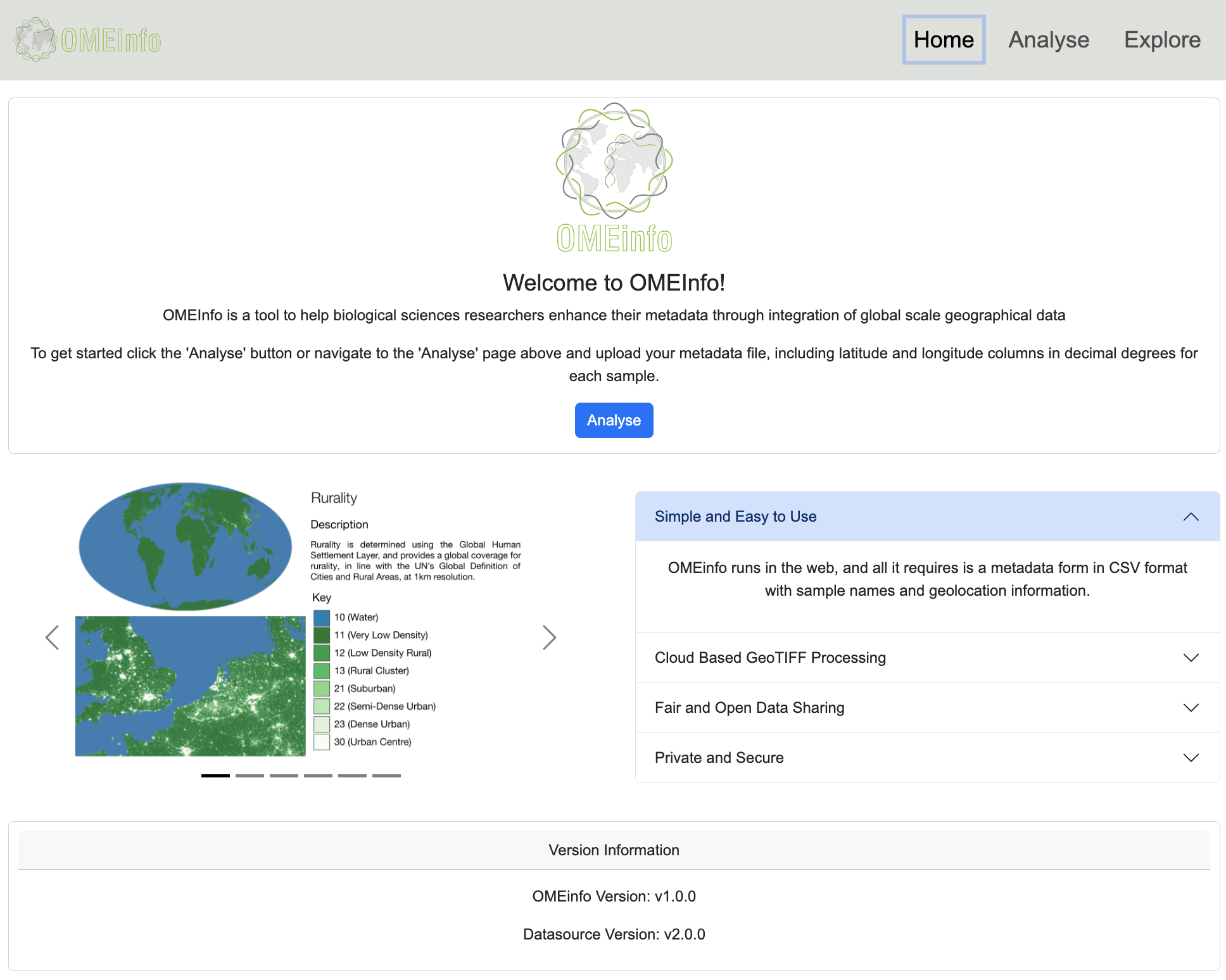


1. From the analyse page, either drag and drop or click through to select the file for analysis. The file should be in CSV or TSV format, and contain at a minimum a sample name, latitude and longitude. Latitude and longitude should be provided in EPSG:4326 format. Once uploaded, OMEinfo will begin processing the file, and a progress wheel will be displayed.


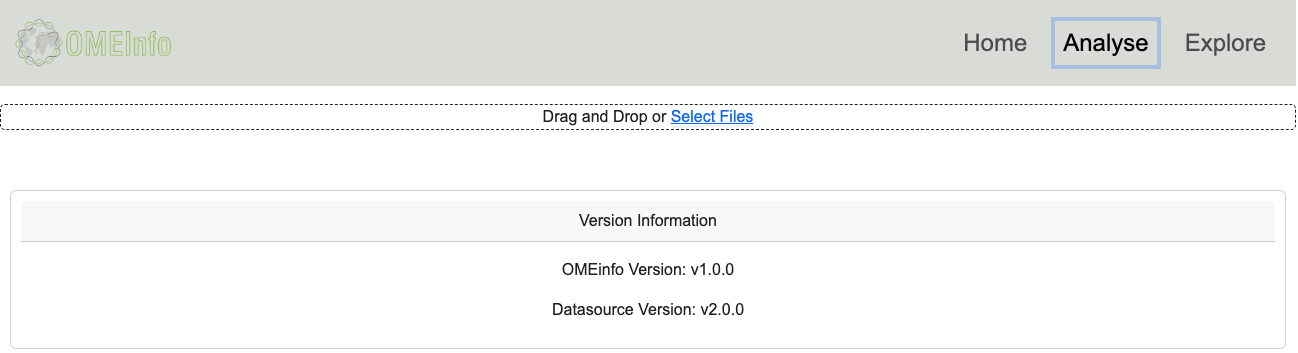

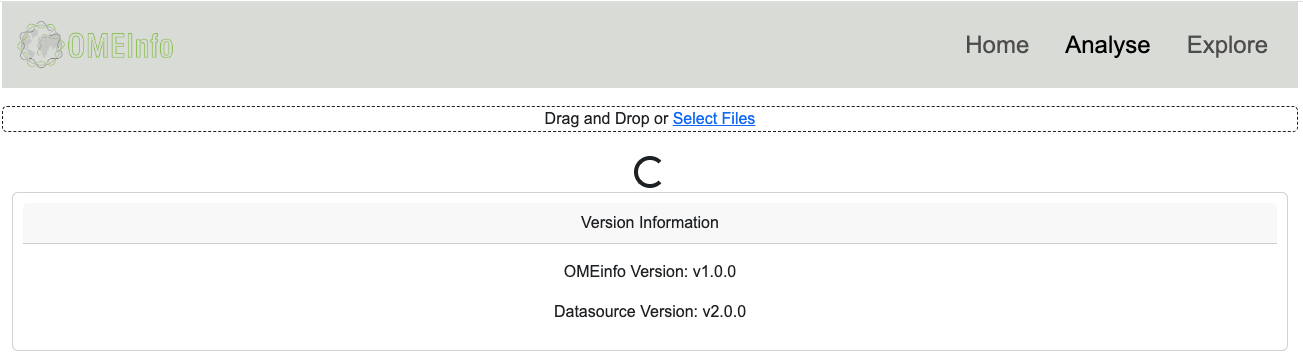


1. Once analysis has completed, the data visualisation section will be presented. Three different interactive views of the data are available, “graph” displays histograms and bar charts (depending on data type) of the annotated metadata, “map” allows users to investigate annotated properties in relation to their geographic location (full annotation is presented for a sample upon hover-over), and “table” allows users to view the annotated data underpinning both of these visualisations.


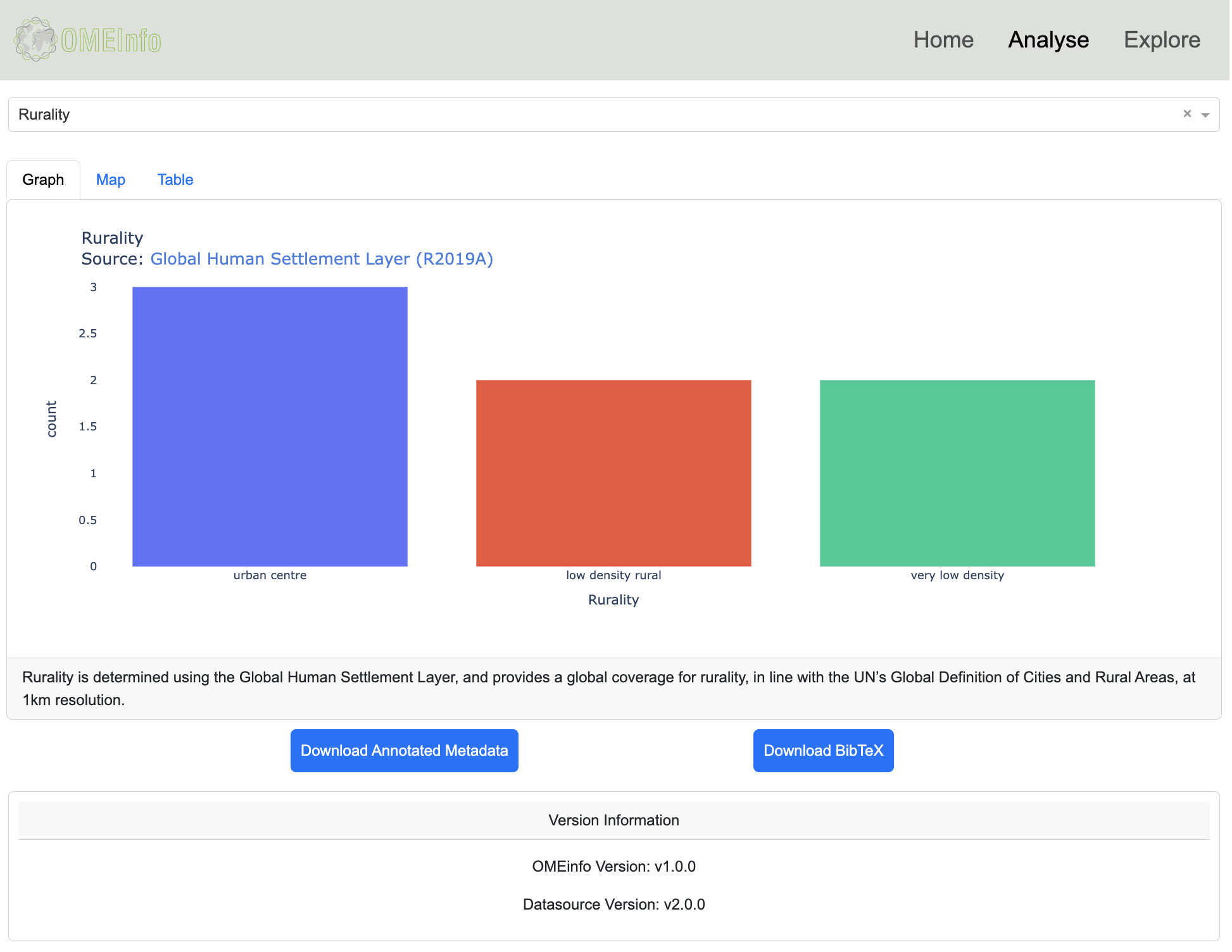

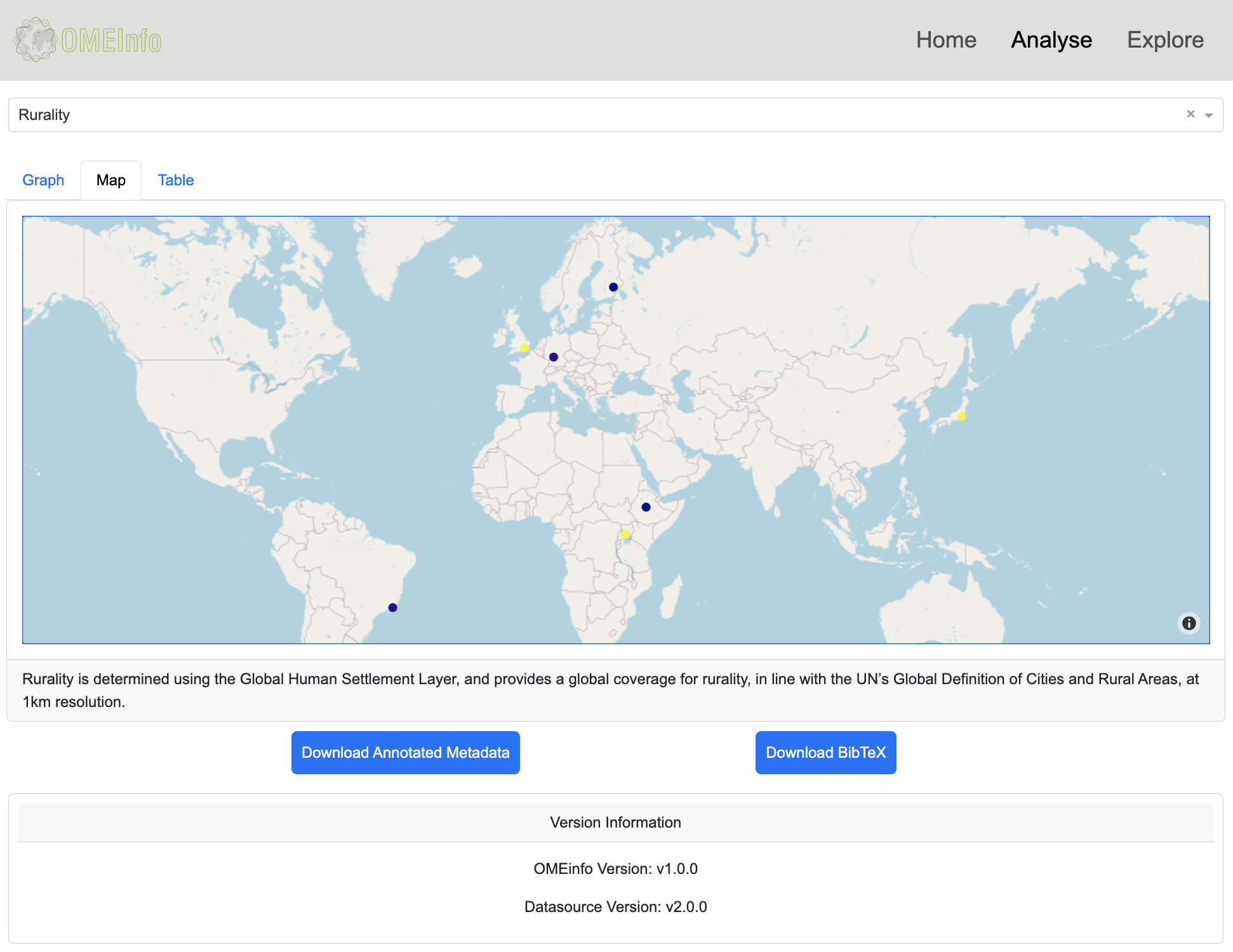

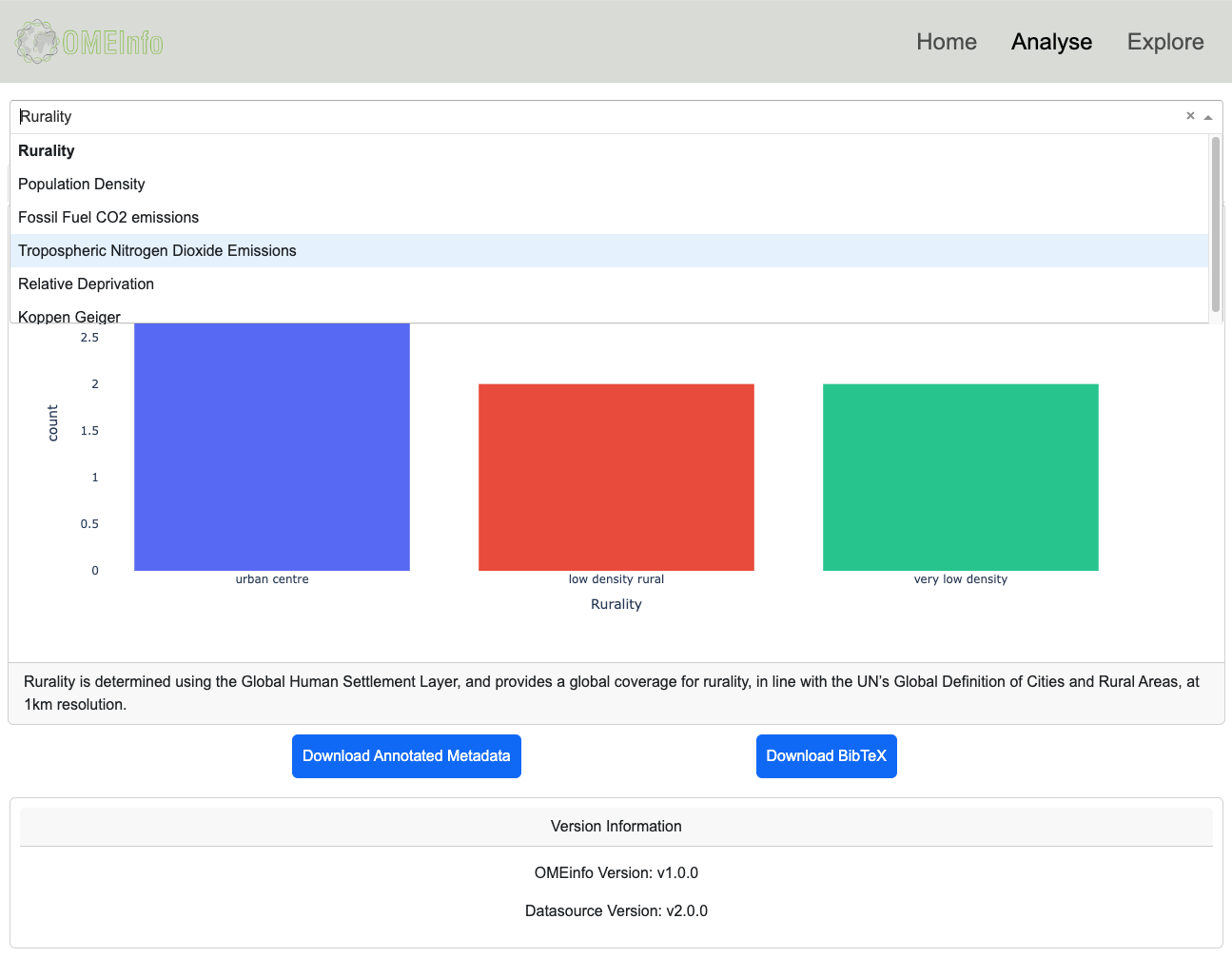


The data focus can be selected using the drop-down menu above the visualisations (default = Rurality).

1. Users can then download their annotated metadata as a TSV file using the download button. The download BibTeX button provides users with a list of citations necessary for properly citing all data sources used within OMEinfo for annotation.

### **Supplementary Information Section 2B:** a walkthrough of the OMEinfo workflow using the command line interface.

The OMEinfo CLI tool allows for rapid annotation of geographic metadata without the additional visualisations and data explorations available in the Dash-app. This tool is suited for large-scale processing of samples, and has been tested on datasets of >5 million samples. To run OMEinfo CLI with the sample data provided in the software repository:

1. Run the following command from the same location as the test file:

omeinfo.py --location_file test_addresses.tsv

1. OMEinfo CLI will provide a summary of the file to be analysed, and begin processing samples.


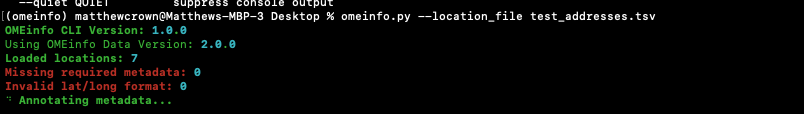


1. Following analysis completion, the annotated metadata will be saved as a TSV format file, and the BibTeX citations necessary for proper citation of the underlying data sources will be saved. The default name for the annotated files is “annotated_locations.tsv”, this can be changed using the “--output_file” flag.
2. In addition to automatically saving the annotated locations file, OMEinfo CLI will also provide a summary of the output


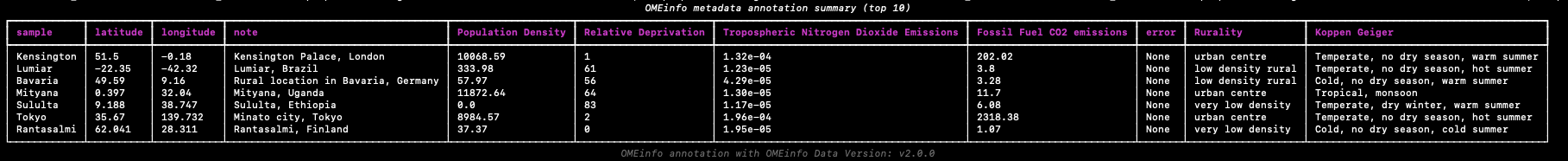


This can be controlled using the flags “--quiet” to provide no summary output, or “--n_samples” to specify the number of table rows you wish to view in the command line.

### **Supplementary Information Section 3:** benchmarking of large sample sizes with OMEinfo on samples from the ENA

To prepare the OMEinfo ENA and OMEinfo ENA Env datasets, metadata for samples in the ENA was accessed using the advanced search function of the ENA browser. This generated a cURL command which was then executed to download all matching sequences:

curl -X POST -H "Content-Type: application/x-www-form-urlencoded" -d 'result=sample&query=environmental_sample%3DTRUE%20%26%20geo_loc(-90%2C180%2C90%2C180)&fields=altitude%2Cassembly_quality%2Cassembly_software%2Cbinning_software%2Cbio_material%2Cbroad_scale_environmental_context%2Cbroker_name%2Ccell_line%2Ccell_type%2Ccenter_name%2Cchecklist%2Ccollected_by%2Ccollection_date%2Ccollection_date_end%2Ccollection_date_start%2Ccompleteness_score%2Ccontamination_score%2Ccountry%2Ccultivar%2Cculture_collection%2Cdatahub%2Cdepth%2Cdescription%2Cdev_stage%2Cecotype%2Celevation%2Cenvironment_biome%2Cenvironment_feature%2Cenvironment_material%2Cenvironmental_medium%2Cenvironmental_sample%2Cexperimental_factor%2Cfirst_public%2Cgermline%2Chost%2Chost_body_site%2Chost_genotype%2Chost_gravidity%2Chost_growth_conditions%2Chost_phenotype%2Chost_scientific_name%2Chost_sex%2Chost_status%2Chost_tax_id%2Cidentified_by%2Cinvestigation_type%2Cisolate%2Cisolation_source%2Ckeywords%2Clast_updated%2Clat%2Clocal_environmental_context%2Clocation%2Clocation_end%2Clocation_start%2Clon%2Cmarine_region%2Cmating_type%2Cncbi_reporting_standard%2Cph%2Cproject_name%2Cprotocol_label%2Csalinity%2Csample_accession%2Csample_alias%2Csample_capture_status%2Csample_collection%2Csample_description%2Csample_material%2Csample_title%2Csampling_campaign%2Csampling_platform%2Csampling_site%2Cscientific_name%2Csecondary_sample_accession%2Csequencing_method%2Cserotype%2Cserovar%2Csex%2Cspecimen_voucher%2Cstatus%2Cstrain%2Cstudy_accession%2Csub_species%2Csub_strain%2Csubmission_tool%2Csubmitted_host_sex%2Ctag%2Ctarget_gene%2Ctax_id%2Ctaxonomic_classification%2Ctaxonomic_identity_marker%2Ctemperature%2Ctissue_lib%2Ctissue_type%2Cvariety&format=tsv' "https://www.ebi.ac.uk/ena/portal/api/search" -o ena_all_171023.tsv

From a total of 34,716,112 samples queried [access date: 2023-10-13], 5,361,056 samples had an associated latitude and longitude making them suitable for processing with OMEinfo. This represents an ideal large-scale dataset for benchmarking the performance of OMEinfo, as well as providing an opportunity to distribute a precomputed OMEinfo dataset well suited to meta-analysis of existing studies which further improves the availability of globally consistent geographic metadata.

Through analysis with OMEinfo CLI v1 and a local copy of the OMEinfo v2 dataset, we were able to annotate the metadata of 5,338,430 samples in ENA with geographical metadata (22,626 samples contained invalid latitude/longitude data) in less than 30 minutes single-threaded (Intel Xeon Gold 6230 CPU @ 2.10GHz, ​​Rocky Linux release 8.8). An additional dataset was produced covering samples specifically marked as environmental (metadata feature describes samples derived from an environmental DNA sample) in ENA. A total of 190,573 samples are marked as environmental, with 135,517 containing valid coordinates for analysis. The total analysis time for this dataset (same parameters as full) took 60.27 seconds.

These annotation files can be downloaded from the FigShare (DOI: <https://doi.org/10.6084/m9.figshare.25003217>).

### **Table S1.**Varying sources of metadata in existing microbial ecology studies.

| **Study** | **Geospatial Info (Source)** | **Metadata Feature Issue** | **Reference** |
| --- | --- | --- | --- |
| Geography and Location Are the Primary Drivers of Office Microbiome Composition | Köppen-Geiger climate classification (Unspecified) | Source of climate classification unspecified. | [(Chase *et al.*, 2016)](https://paperpile.com/c/zaN4J5/RzDV) |
| Urbanization pressures alter tree rhizosphere microbiomes | Rurality/Urbanisation (US Census Bureau) | Uses a national-level definition of rurality. | [(Rosier *et al.*, 2021)](https://paperpile.com/c/zaN4J5/kXUA) |
| Continental-scale distributions of dust-associated bacteria and fungi | Location | 1-degree resolution latitude/longitude - approximately 11.1km resolution. | [(Barberán *et al.*, 2015)](https://paperpile.com/c/zaN4J5/x8bN) |
| Farm-like indoor microbiota in non-farm homes protects children from asthma development | Rurality and location | Rurality reported without methodology. Latitude/longitude is only available on request. | [(Kirjavainen *et al.*, 2019)](https://paperpile.com/c/zaN4J5/CJEm) |
| Home Life: Factors structuring the bacterial diversity found within and between homes | Location | Location is constant for 40 homes in a 1000km^2^ region. | [(Dunn *et al.*, 2013)](https://paperpile.com/c/zaN4J5/oPCm) |
| Microbial exposures in moisture-damaged schools and associations with respiratory symptoms in students: A multi-country environmental exposure study | Rurality and location | Building setting reported but the method used is not described. Sample location reported as nearest city, with no latitude or longitude. | [(Adams *et al.*, 2021)](https://paperpile.com/c/zaN4J5/ssyg) |
| Urbanization Reduces Transfer of Diverse Environmental Microbiota Indoors | Rurality and location | Constant latitude/longitude reported for 26 urban and 30 rural homes. Rurality uses a national-level definition. | [(Parajuli *et al.*, 2018)](https://paperpile.com/c/zaN4J5/NryL) |
| Exposure to farming in early life and development of asthma and allergy: a cross-sectional survey | Rurality | Population density and farming characteristics considered for region similarity, but data sources and methodology not described. | [(Riedler *et al.*, 2001)](https://paperpile.com/c/zaN4J5/LWdS) |
| Environmental factors shaping the gut microbiome in a Dutch population | Rurality (Statistics Netherlands) | Uses a national-level definition of rurality. | [(Gacesa *et al.*, 2022)](https://paperpile.com/c/zaN4J5/TCe2) |

### **Table S2.**A sample of the different definitions of rurality in use across the world.

| **Country** | **Urban/Rural Definition** | **Reference** |
| --- | --- | --- |
| Canada | An urban area was defined as having a population of at least 1,000 and a density of 400 or more people per square kilometre | [(Government of Canada and Canada, 2018)](https://paperpile.com/c/zaN4J5/Z2eeB) |
| USA | ~2500 inhabitants/km^2^(1000/mile^2^) | [(Cromartie, no date)](https://paperpile.com/c/zaN4J5/69CjO) |
| Japan | Define “Densely Inhabited Districts” as those with 4000+ inhabitants/km^2^ | [(Statistics Bureau, Ministry of Internal Affairs and Communications, no date)](https://paperpile.com/c/zaN4J5/Glbtg) |
| UK | Rural defined as areas which “fall outside of settlements with more than 10,000 resident population” | [(UK Government, 2017)](https://paperpile.com/c/zaN4J5/fJ3w7) |
| Iceland | “Rural areas are agricultural areas or villages with less than 200 inhabitants” | [(*Statistics Iceland: Fewer than 6% of the population live in rural areas*, no date)](https://paperpile.com/c/zaN4J5/kV6o3) |
| Norway | Urban defined as minimum 200 inhabitants per cluster of buildings. Minimum population density in urban settlements (2022) = 236/km^2^ | [(*Population and land area in urban settlements*, no date)](https://paperpile.com/c/zaN4J5/rxUhX) |

###

### **Table S3.**OMEinfo v2 data sources

| **Annotation** | **Source** | **Update Frequency** | **Licence** | **Reference** |
| --- | --- | --- | --- | --- |
| Rurality and Population Density | Global Human Settlement Layer | New versions of the dataset are released periodically with unclear schedule. As new versions become available, they will be integrated into OMEinfo data packet. | CC BY 4.0 | [(Joint Research Centre (European Commission) *et al.*, 2019)](https://paperpile.com/c/zaN4J5/ws4b) |
| Fossil Fuel CO_2_ Emissions | ODIAC | New versions are released annually. As new versions become available they will be validated and integrated into OMEinfo data packet. | CC BY 4.0 | [(Oda and Maksyutov, 2015)](https://paperpile.com/c/zaN4J5/3QrB) |
| Tropospheric NO_2_ Emissions | Copernicus Sentinel S5p | New measurements released daily. New annual measurements will be integrated into OMEinfo yearly following validation. | (see <https://sentinel.esa.int/documents/247904/690755/sentinel_data_legal_notice> ) | [(Romahn *et al.*, 2022)](https://paperpile.com/c/zaN4J5/FQr0) |
| Köppen-Geiger Climate Classification | Beck *et al*. | No update planned, sourced from academic publication. If updated data becomes available we will integrate into future releases. | CC BY 4.0 | [(Beck *et al.*, 2018)](https://paperpile.com/c/zaN4J5/Jsdx) |
| Relative Deprivation | SEDAC | No public update schedule from NASA SEDAC, should updated data become available we will integrate into future release. | CC BY 4.0 | [(Center for International Earth Science Information Network - CIESIN - Columbia University, 2022)](https://paperpile.com/c/zaN4J5/XOwm) |

### **Table S4.**List of software and versions used in OMEinfo (Dash App and CLI)

| **Software** | **Usage** | **OMEinfo version** | **Version** | **Reference** |
| --- | --- | --- | --- | --- |
| rio-cogeo | Data preprocessing, querying COG format files. Depends on both Rasterio and GDAL. | App + CLI | v5.0 | [(*rio-cogeo: v5.0.0*, no date)](https://paperpile.com/c/zaN4J5/cCsoA) |
| Geospatial Data Abstraction Library (GDAL) | Data preprocessing, querying COG format files. | App + CLI | v3.7.0 | [(GDAL/OGR contributors, 2023)](https://paperpile.com/c/zaN4J5/UnZlj) |
| Rasterio | Data preprocessing, querying COG format files. | App + CLI | v1.3.7 | [(Gillies and Others, 2013-)](https://paperpile.com/c/zaN4J5/MYPRY) |
| Dash-Bootstrap | Web-app development | App | v1.4.1 | [(*dash-bootstrap-components: v1.4.1*, no date)](https://paperpile.com/c/zaN4J5/ZneMA) |
| Rich | Annotated data visualisation | CLI | v13.6.0 | [(*rich: v13.6.0*, no date)](https://paperpile.com/c/zaN4J5/SLTdb) |
| Plotly Python | Annotated data visualisation | App | v5.17.0 | [(*plotly.py: v5.17.0*, no date)](https://paperpile.com/c/zaN4J5/zxnM3) |
| QGIS | Digital Elevation Model representation of underlying data sources. | App | v3.28.2 | [(QGIS Development Team, no date)](https://paperpile.com/c/zaN4J5/ajZev) |
| Qgis2threejs | Digital Elevation Model visualisation | App | v2.7.1 | [(Akagi, no date)](https://paperpile.com/c/zaN4J5/CpScG) |
| Dash | Web-app development | App | v2.9.3 | [(*dash: v2.9.3*, no date)](https://paperpile.com/c/zaN4J5/0dYrU) |

**
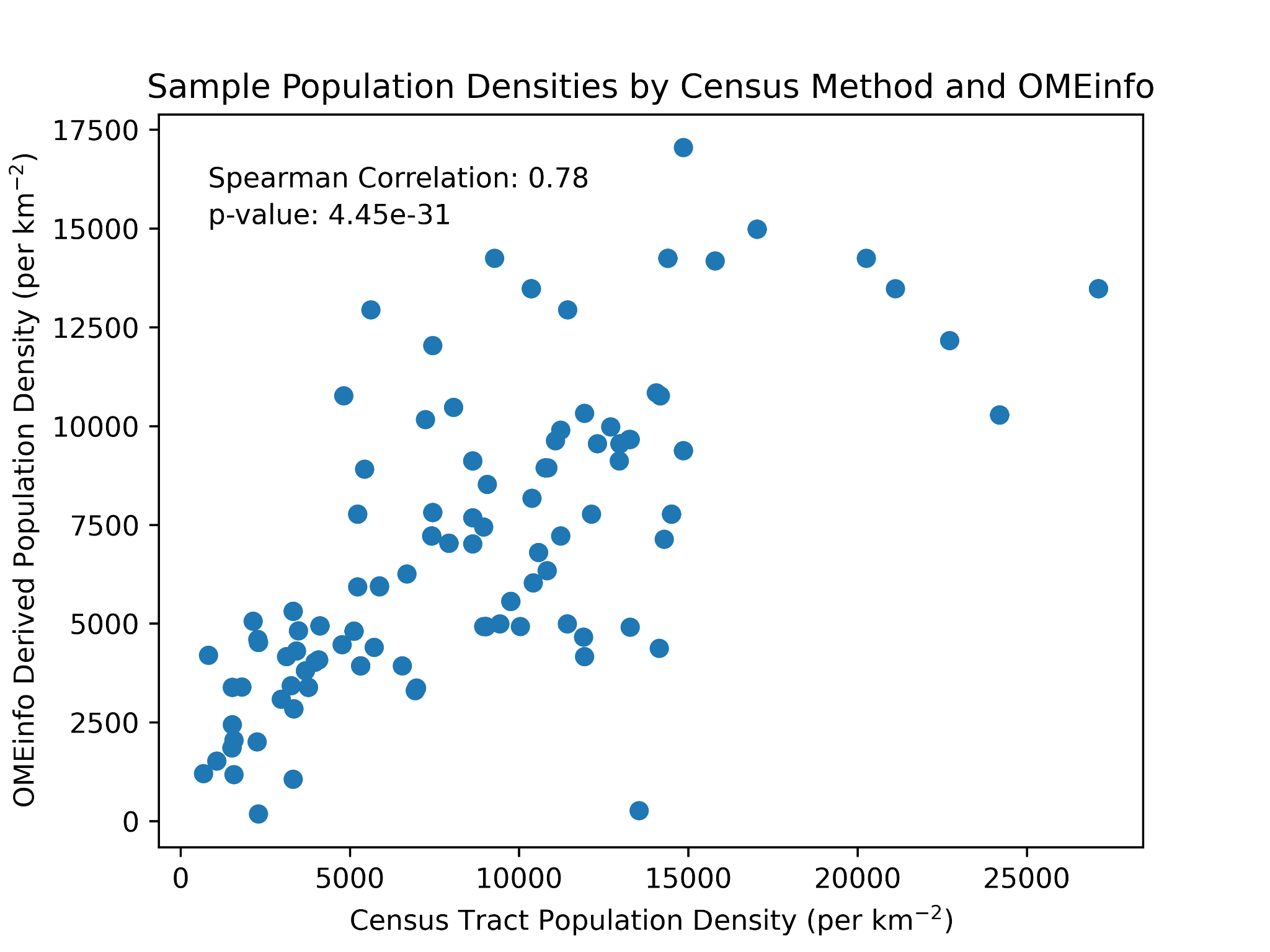
**

### **Fig. S1.**: Correlation between census-tract derived from Wang et al.[(Wang *et al.*, 2018)](https://paperpile.com/c/zaN4J5/Jlms) using data provided by the corresponding author and OMEinfo derived population densities.

###
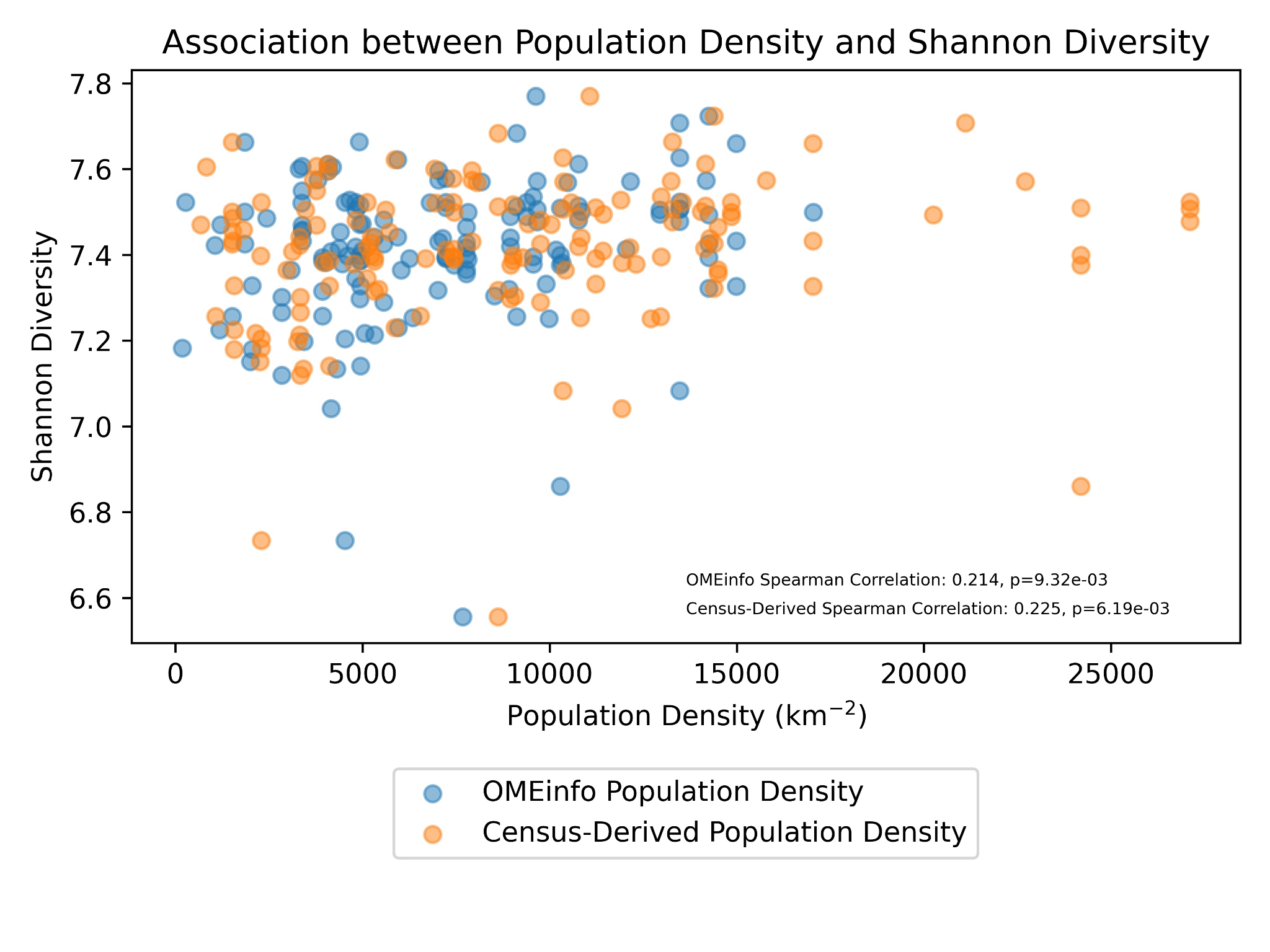


### **Fig. S2.**: Relationship between population density and Shannon diversity, using OMEinfo derived population density, and census-derived population density (provided by the corresponding authors of [(Wang *et al.*, 2018)](https://paperpile.com/c/zaN4J5/Jlms)). The plot shows OMEinfo provides a functional, globally consistent stand-in for census-tract derived data, as demonstrated by replication of the previously published Spearman correlation coefficient for population density in [(Wang *et al.*, 2018)](https://paperpile.com/c/zaN4J5/Jlms).

## References

[Adams, R.I. *et al.* (2021) ‘Microbial exposures in moisture-damaged schools and associations with respiratory symptoms in students: A multi-country environmental exposure study’, *Indoor air*, 31(6), pp. 1952–1966.](http://paperpile.com/b/zaN4J5/ssyg)

[Akagi, M. (no date) *Qgis2threejs: v2.7.1*. Github. Available at:](http://paperpile.com/b/zaN4J5/CpScG) <https://github.com/minorua/Qgis2threejs> [(Accessed: 13 October 2023).](http://paperpile.com/b/zaN4J5/CpScG)

[Barberán, A. *et al.* (2015) ‘Continental-scale distributions of dust-associated bacteria and fungi’, *Proceedings of the National Academy of Sciences of the United States of America*, 112(18), pp. 5756–5761.](http://paperpile.com/b/zaN4J5/x8bN)

[Beck, H. *et al.* (2018) ‘Present and future Köppen-Geiger climate classification maps at 1-km resolution’, *Scientific Data*, 5, p. 180214.](http://paperpile.com/b/zaN4J5/Jsdx)

[Center for International Earth Science Information Network - CIESIN - Columbia University (2022) ‘Global Gridded Relative Deprivation Index (GRDI), Version 1’. Palisades, New York: NASA Socioeconomic Data and Applications Center (SEDAC). Available at: https://doi.org/](http://paperpile.com/b/zaN4J5/XOwm)[10.7927/3xxe-ap97](http://dx.doi.org/10.7927/3xxe-ap97)[.](http://paperpile.com/b/zaN4J5/XOwm)

[Chase, J. *et al.* (2016) ‘Geography and Location Are the Primary Drivers of Office Microbiome Composition’, *mSystems*. Edited by J.A. Gilbert, 1(2), pp. mSystems.00022–16, e00022–16.](http://paperpile.com/b/zaN4J5/RzDV)

[Cromartie, J. (no date) *What is Rural?* Available at:](http://paperpile.com/b/zaN4J5/69CjO) <https://www.ers.usda.gov/topics/rural-economy-population/rural-classifications/what-is-rural/> [(Accessed: 13 October 2023).](http://paperpile.com/b/zaN4J5/69CjO)

[*dash-bootstrap-components: v1.4.1* (no date). Github. Available at:](http://paperpile.com/b/zaN4J5/ZneMA) <https://github.com/facultyai/dash-bootstrap-components> [(Accessed: 13 October 2023).](http://paperpile.com/b/zaN4J5/ZneMA)

[*dash: v2.9.3* (no date). Github. Available at:](http://paperpile.com/b/zaN4J5/0dYrU) <https://github.com/plotly/dash> [(Accessed: 13 October 2023).](http://paperpile.com/b/zaN4J5/0dYrU)

[Dunn, R.R. *et al.* (2013) ‘Home life: factors structuring the bacterial diversity found within and between homes’, *PloS one*. Edited by S. Bertilsson, 8(5), p. e64133.](http://paperpile.com/b/zaN4J5/oPCm)

[Gacesa, R. *et al.* (2022) ‘Environmental factors shaping the gut microbiome in a Dutch population’, *Nature*, 604(7907), pp. 732–739.](http://paperpile.com/b/zaN4J5/TCe2)

[GDAL/OGR contributors (2023) ‘GDAL/OGR Geospatial Data Abstraction software Library’. Open Source Geospatial Foundation. Available at: https://doi.org/](http://paperpile.com/b/zaN4J5/UnZlj)[10.5281/zenodo.5884351](http://dx.doi.org/10.5281/zenodo.5884351)[.](http://paperpile.com/b/zaN4J5/UnZlj)

[Gillies, S. and Others (2013-) *Rasterio: v1.3.8.post2*. Mapbox. Available at:](http://paperpile.com/b/zaN4J5/MYPRY) <https://github.com/rasterio/rasterio>[.](http://paperpile.com/b/zaN4J5/MYPRY)

[Government of Canada and Canada, S. (2018) *Population Centre and Rural Area Classification 2016 - Definitions*. Available at:](http://paperpile.com/b/zaN4J5/Z2eeB) <https://www.statcan.gc.ca/en/subjects/standard/pcrac/2016/definitions> [(Accessed: 13 October 2023).](http://paperpile.com/b/zaN4J5/Z2eeB)

[Joint Research Centre (European Commission) *et al.* (2019) *GHSL data package 2019: public release GHS P2019*. LU: Publications Office of the European Union.](http://paperpile.com/b/zaN4J5/ws4b)

[Kirjavainen, P.V. *et al.* (2019) ‘Farm-like indoor microbiota in non-farm homes protects children from asthma development’, *Nature medicine*, 25(7), pp. 1089–1095.](http://paperpile.com/b/zaN4J5/CJEm)

[Oda, T. and Maksyutov, S. (2015) ‘ODIAC Fossil Fuel CO2 Emissions Dataset’, *Center for Global Environmental Research, National Institute for Environmental Studies* [Preprint]. Available at: https://doi.org/](http://paperpile.com/b/zaN4J5/3QrB)[10.17595/20170411.001](http://dx.doi.org/10.17595/20170411.001)[.](http://paperpile.com/b/zaN4J5/3QrB)

[Parajuli, A. *et al.* (2018) ‘Urbanization Reduces Transfer of Diverse Environmental Microbiota Indoors’, *Frontiers in microbiology*, 9, p. 84.](http://paperpile.com/b/zaN4J5/NryL)

[*plotly.py: v5.17.0* (no date). Github. Available at:](http://paperpile.com/b/zaN4J5/zxnM3) <https://github.com/plotly/plotly.py> [(Accessed: 15 January 2024).](http://paperpile.com/b/zaN4J5/zxnM3)

[*Population and land area in urban settlements* (no date) *SSB*. Available at:](http://paperpile.com/b/zaN4J5/rxUhX) <https://www.ssb.no/en/befolkning/folketall/statistikk/tettsteders-befolkning-og-areal> [(Accessed: 13 October 2023).](http://paperpile.com/b/zaN4J5/rxUhX)

[QGIS Development Team (no date) *QGIS Geographic Information System v3.28.2*. QGIS Association. Available at:](http://paperpile.com/b/zaN4J5/ajZev) <https://www.qgis.org>[.](http://paperpile.com/b/zaN4J5/ajZev)

[*rich: v13.6.0* (no date). Github. Available at:](http://paperpile.com/b/zaN4J5/SLTdb) <https://github.com/Textualize/rich> [(Accessed: 13 October 2023).](http://paperpile.com/b/zaN4J5/SLTdb)

[Riedler, J. *et al.* (2001) ‘Exposure to farming in early life and development of asthma and allergy: a cross-sectional survey’, *The Lancet*, 358(9288), pp. 1129–1133.](http://paperpile.com/b/zaN4J5/LWdS)

[*rio-cogeo: v5.0.0* (no date). Github. Available at:](http://paperpile.com/b/zaN4J5/cCsoA) <https://github.com/cogeotiff/rio-cogeo> [(Accessed: 13 October 2023).](http://paperpile.com/b/zaN4J5/cCsoA)

[Romahn, F. *et al.* (2022) *Sentinel-5 Precursor/TROPOMI Level 2 Product User Manual: Cloud Properties*. European Space Agency (ESA).](http://paperpile.com/b/zaN4J5/FQr0)

[Rosier, C.L. *et al.* (2021) ‘Urbanization pressures alter tree rhizosphere microbiomes’, *Scientific reports*, 11(1), p. 9447.](http://paperpile.com/b/zaN4J5/kXUA)

[Statistics Bureau, Ministry of Internal Affairs and Communications (no date) *What is a Densely Inhabited District?* Available at:](http://paperpile.com/b/zaN4J5/Glbtg) <https://www.stat.go.jp/english/data/chiri/did/1-1.html> [(Accessed: 13 October 2023).](http://paperpile.com/b/zaN4J5/Glbtg)

[*Statistics Iceland: Fewer than 6% of the population live in rural areas* (no date) *Statistics Iceland*. Available at:](http://paperpile.com/b/zaN4J5/kV6o3) <https://statice.is/publications/news-archive/inhabitants/population-by-urban-nuclei-2001-2020/> [(Accessed: 13 October 2023).](http://paperpile.com/b/zaN4J5/kV6o3)

[UK Government (2017) ‘Defining Rural Areas’. Available at:](http://paperpile.com/b/zaN4J5/fJ3w7) <https://assets.publishing.service.gov.uk/government/uploads/system/uploads/attachment_data/file/597751/Defining_rural_areas__Mar_2017_.pdf>[.](http://paperpile.com/b/zaN4J5/fJ3w7)

[Wang, H. *et al.* (2018) ‘Soil Bacterial Diversity Is Associated with Human Population Density in Urban Greenspaces’, *Environmental science & technology*, 52(9), pp. 5115–5124.](http://paperpile.com/b/zaN4J5/Jlms)
